# A promoting early presentation intervention increases breast cancer awareness in older women after 2 years: a randomised controlled trial

**DOI:** 10.1038/bjc.2011.205

**Published:** 2011-06-07

**Authors:** L J L Forbes, L Linsell, L Atkins, C Burgess, L Tucker, L Omar, A J Ramirez

**Affiliations:** 1 Promoting Early Presentation Group, Kings College London, Adamson Centre for Mental Health, South Wing, St Thomas' Hospital, London SE1 7EH, UK

**Keywords:** aged, awareness, breast cancer, complex intervention, delayed presentation, randomized controlled trials

## Abstract

**Background:**

We have developed the Promoting Early Presentation (PEP) Intervention to equip older women with the knowledge, skills, confidence and motivation to present promptly with breast symptoms, and thereby improve survival from breast cancer. The PEP Intervention consists of a 10-min interaction between a radiographer and an older woman, supported by a booklet. Our previous report showed that at 1 year, the PEP intervention increased the proportion who were breast cancer aware compared with usual care.

**Methods:**

We randomised 867 women aged 67–70 years attending for their final routine appointment on the National Health Service Breast Screening Programme to receive the PEP Intervention, a booklet alone or usual care. The primary outcome was breast cancer awareness measured using a validated questionnaire asking about knowledge of breast cancer symptoms, knowledge that the risk of breast cancer increases with age and breast checking behaviour.

**Results:**

At 2 years, the PEP Intervention increased the proportion who were breast cancer aware compared with usual care (21 *vs* 6% odds ratio 8.1, 95% confidence interval 2.7–25.0).

**Conclusions:**

The uniquely large and sustained effect of the PEP Intervention on breast cancer awareness increases the likelihood that a woman will present promptly should she develop breast cancer symptoms up to many years later.

Delay in diagnosis leads to poorer survival in breast cancer (Richards *et al*, 1999). Delay in diagnosis may be due to delayed presentation by some women or delayed onward referral by primary care physicians. Older women are more likely to delay presentation in breast cancer (Ramirez *et al*, 1999), present with more advanced disease, and have much higher short-term mortality rates than younger women (Moller *et al*, 2010). Delay in presentation is likely to be due to poor awareness of symptoms and negative beliefs about breast cancer and its treatment (Ramirez *et al*, 1999; Grunfeld *et al*, 2002).

Promoting early presentation in women attending for their final round of breast screening, at whatever age that may be, may reduce stage at diagnosis cost-effectively among older women. It is unlikely to lead to overdiagnosis: a very high proportion of older women referred to symptomatic breast clinics are subsequently diagnosed with breast cancer (Wishart *et al*, 2010).

We have developed an intervention to equip older women with breast cancer awareness (Burgess *et al*, 2008), in other words, the knowledge, confidence, motivation and skills to present promptly to primary care on discovering breast cancer symptoms, and thereby improve survival. We have carried out a randomised controlled trial of this intervention delivered when women attended for final routine mammogram at age 67–70 years on the UK National Health Service Breast Screening Programme. At 1 year, the intervention increased the proportion of women who were breast cancer aware more than any other intervention of its kind (Austoker *et al*, 2009; Linsell *et al*, 2009). Continued follow-up is important because a woman needs to retain the knowledge, confidence, motivation and skills to present promptly; it may be many years before she develops a breast symptom that may be due to breast cancer. We report here the 2-year results of the trial. The intervention is now known as the Promoting Early Presentation (PEP) Intervention.

## Material and Methods

The PEP Intervention is a scripted 10-min one-to-one interaction between a radiographer and the woman, supported by a booklet, delivered after the woman has had her mammographic examination. The script covers the symptoms of breast cancer, the increased risk of developing breast cancer with increasing age, rehearsal of the skills required to check for breast changes and what women should do if they discover a breast change.

We randomised women attending their final routine appointment on the National Health Service Breast Screening Programme to the PEP Intervention, or booklet alone, or usual care, in three breast screening services in London and South East England during 2007–2008. Usual care consisted of standard practice in the National Health Service Breast Screening Programme after final invited mammogram, in other words, brief verbal and/or written information to each woman that she would not longer be invited for screening every 3 years, but that she might continue to be screened at this interval on request.

The primary outcome was breast cancer awareness measured using a self-complete postal questionnaire (Linsell *et al*, 2010) asking about knowledge that the risk of breast cancer increases with age (*‘In the next year who is most likely to get breast cancer?’* A 30-year-old woman; a 50-year-old woman; a 70-year-old woman; a woman of any age), recognition of non-lump symptoms of breast cancer from a list and reported frequency of breast checking (*‘How often do you check your breasts?’* Rarely or never; at least every 6 months; at least once a month; at least once a week.).

We measured breast cancer awareness at baseline, 1 month, 6 months, 1 year and 2 years post randomisation. We analysed the data using robust generalized estimating equations with a logit link and binomial distribution for the outcome (Zeger and Liang, 1986) using Stata version 11 (StataCorp, College Station, TX, USA). This method takes account of the correlation between repeated observations of the same individual. We performed an unadjusted analysis and repeated it adjusting for baseline characteristics: marital status, education, ethnicity and Index of Multiple Deprivation.

Our previous report provides further detail of the methods (Linsell *et al*, 2009).

## Results

**Figure 1:**
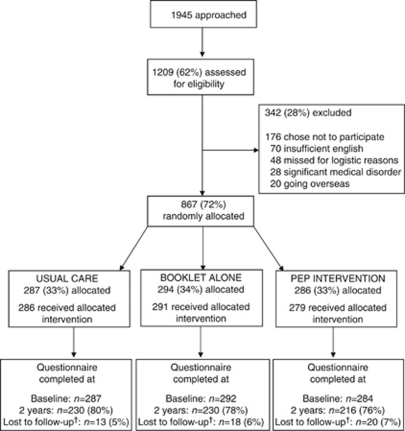
Flow of participants through trial. ^†^Lost to follow-up: withdrew from the trial, died or moved away without giving forwarding address.

We randomised 867 women to receive usual care (*n*=287), the booklet alone (*n*=294) or the PEP Intervention (*n*=286; Figure 1). We received breast cancer awareness questionnaires from 78% at 2 years; response rates were similar in each arm (Figure 1). Fifty-one women (6%) were lost to follow-up.

Women who responded at 2 years were more likely to be married or cohabiting and to be of white British ethnic group, and lived in less deprived areas than women who did not respond (Table 1). There was no conclusive evidence that women who responded at 2 years had better breast cancer awareness at baseline. The differences between responders and non-responders for the three components of breast cancer awareness were inconsistent and of borderline significance.

**Figure 2:**
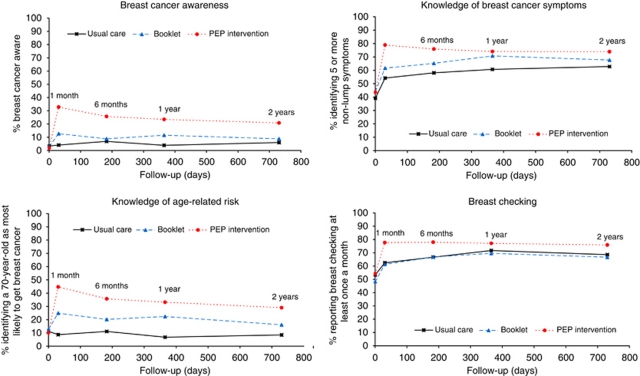
Breast cancer awareness and component items over follow-up.

Table 2 shows breast cancer awareness and its components at baseline and 2 years in the three arms of the trial. Figure 2 shows the change in breast cancer awareness score and its components at baseline, 1 month, 6 months, 1 year and 2 years. The PEP Intervention increased the proportions who were breast cancer aware at 2 years compared with usual care, and the booklet alone did not. Knowledge of non-lump symptoms, knowledge that the risk of breast cancer increased with age and reported breast checking increased in the PEP Intervention and booklet arms over follow-up. However, knowledge of non-lump symptoms and reported breast checking, but not knowledge of age-related risk, also increased in the usual care arm.

In the analysis adjusting for baseline characteristics, the odds ratios (ORs) for breast cancer awareness for the booklet *vs* usual care and the PEP Intervention *vs* usual care were larger (booklet *vs* usual care: OR 2.8, 95% confidence interval (CI) 0.9–9.1; PEP Intervention *vs* usual care: OR 12.2, 95% CI 3.8–38) than for the unadjusted results. The CIs were, however, much wider.

## Discussion

The PEP Intervention increased breast cancer awareness in older women compared with usual care at 2 years: 21% of the women who received the PEP Intervention were breast cancer aware compared with 6% of women who received usual care. Of the three components of breast cancer awareness the PEP Intervention had the most marked effect compared with usual care on knowledge that the risk of breast cancer increases with age.

The size of the effect of the PEP Intervention fell slightly between 1 and 2 years compared with usual care. This may be at least partly due to the increase in breast cancer awareness in the usual care arm between 1 and 2 years. One possible explanation is that the questionnaire itself increased breast cancer awareness (a recognised phenomenon known as the ‘mere measurement’ effect; Godin *et al*, 2008) in the usual care arms more than the intervention arms. Also, media reports or cancer awareness initiatives may have increased breast cancer awareness in the usual care arm. Another possible explanation is differential response between the three arms; women in the usual care arm who returned questionnaires at 2 years were slightly better educated than their counterparts in the booklet or PEP Intervention arms (data not shown).

We have found no published reports of randomised controlled trials of interventions to promote cancer awareness and early presentation that have followed-up the participants for as long as 2 years. None of the trials of similar interventions included in a systematic review followed up the participants for more than 1 year after the final component of the intervention; all found much more modest effects on cancer awareness (Austoker *et al*, 2009).

Prolonged follow-up in trials of interventions of this kind is important because their purpose is not simply to raise cancer awareness for its own sake but to provide people with the knowledge, motivation, confidence and skills to detect and interpret symptoms appropriately and present promptly to primary care. They need to have a sustained effect, because even in older women, it may be many years before symptoms develop.

Strengths of our trial include the high level of participation and the prolonged length of follow-up, combined with a high response to follow-up. We used a validated instrument for measuring breast cancer awareness (Linsell *et al*, 2010).

Now that the PEP Intervention has been shown to increase breast cancer awareness in women attending their final round of breast screening, we plan studies to identify and test models of delivery in women who do not attend and who may be at highest risk of delayed symptomatic presentation.

The PEP Intervention, a 10-min interaction with a health professional plus a booklet, promotes breast cancer awareness in older women more, and for longer, than any other intervention of its kind. The English National Health Service Breast Screening Programme is currently piloting implementation of the PEP Intervention in a number of breast screening services to examine feasibility and costs, and the effect on breast cancer awareness in routine clinical practice. If piloting is successful and it is implemented across the Breast Screening Programme, we can examine whether the gains in breast cancer awareness translate into a reduction in the incidence of advanced cancers (and deaths) from older women no longer covered by the programme.

## Figures and Tables

**Table 1:** Comparison of baseline characteristics of responders and non-responders at 2 years

	**Responded at 2 years (*n*=676)**	**No response at 2 years (*n*=187)**	*P* value*
*Marital status*			
Married or cohabiting	401 (60%)	86 (47%)	0.001
			
*Educational level*			
No formal qualifications	240 (38%)	78 (46%)	0.15
O level or equivalent	196 (31%)	51 (30%)	
A level or equivalent	81 (13%)	13 (8%)	
Degree or above	111 (18%)	28 (16%)	
			
*Ethnic group*			
White British	479 (72%)	90 (49%)	<0.001
White other	57 (9%)	21 (12%)	
Black Caribbean	70 (11%)	41 (23%)	
Other	57 (9%)	30 (16%)	
			
*Index of Multiple Deprivation*			
Median interquartile range; 0 (least) to 100 (most) deprived	16.5 (7.8–28.1)	22.4 (14.6–33.0)	<0.001
			
*Baseline breast cancer awareness*			
Identified five or more non-lump symptoms AND	16/643 (2%)	6/171 (4%)	0.53
identified a 70-year-old woman as most likely to get breast cancer AND			
reported breast checking at least once a month			
			
*Baseline knowledge of breast cancer symptoms*			
Identified five or more non-lump symptoms	293/667 (44%)	66/183 (36%)	0.06
			
*Baseline knowledge that risk increases with age*			
Identified a 70-year-old woman as most likely to get breast cancer	67/653 (10%)	27/174 (16%)	0.05
			
*Baseline reported breast checking*			
Reported breast checking at least once a month	362/672 (54%)	83/185 (45%)	0.03

^*^P values from Pearson's *χ*^2^ test for categorical variables and the Wilcoxon rank sum test for Index of Multiple Deprivation.

**Table 2:** Breast cancer awareness at baseline and 2 years post-randomisation

	**Baseline**			**Two years**		
	**Usual care**	**Booklet**	**PEP Intervention**	**Usual care**	**Booklet**	**PEP Intervention**
*Breast cancer awareness*						
Number (%) breast cancer aware^a^	9/267	8/275	5/272	13/218	19/217	42/202
	(3.4)	(2.9)	(1.8)	(6.0)	(8.8)	(20.8)
Odds ratio^b^ (95% CI), *P* value (*vs* usual care)				1.0	1.8 (0.6–5.3), 0.32	8.1 (2.7–25.0), <0.001
						
*Knowledge of breast cancer symptoms*						
Identified five or more non-lump symptoms (%)	111/284	126/286	122/280	140/223	153/226	156/211
	(39.1)	(44.1)	(43.6)	(62.8)	(67.7)	(73.9)
Odds ratio^b^ (95% CI), *P* value (*vs* usual care)				1.0	1.1 (0.7–1.6), 0.66	1.4 (0.9–2.1), 0.11
						
*Knowledge that risk increases with age*						
Identified a 70-year-old woman as most likely to get breast cancer (%)	30/269	36/282	28/276	19/224	36/223	60/207
	(11.2)	(12.8)	(10.1)	(8.5)	(16.1)	(29.0)
Odds ratio^b^ (95% CI), *P* value (*vs* usual care)				1.0	1.8 (0.9–3.5), 0.08	4.8 (2.6–9.0), <0.001
						
*Breast checking*						
Reported breast checking at least once a month (%)	152/285	139/288	154/284	157/229	152/228	164/216
	(53.3)	(48.3)	(54.2)	(68.6)	(66.7)	(75.9)
Odds ratio^b^ (95% CI), *P* value (*vs* usual care)				1.0	1.1 (0.8–1.6), 0.54	1.3 (0.9–1.9), 0.14

Abbreviation: CI=confidence interval.

a A woman was considered breast cancer aware if she: identified at least five non-lump symptoms; identified that a 70-year-old woman is most at risk of breast cancer (rather than a 30-year-old, a 50-year-old or a woman of any age); and reported checking her breasts at least once a month.

b Crude odds ratios – not adjusted for baseline characteristics.
